# Sustainable Graphene Production: Flash Joule Heating Utilizing Pencil Graphite Precursors

**DOI:** 10.3390/nano14151289

**Published:** 2024-07-31

**Authors:** Mashhood Zahid, Tomy Abuzairi

**Affiliations:** Department of Electrical Engineering, Faculty of Engineering, Universitas Indonesia, Depok 16424, Indonesia

**Keywords:** flash Joule heating, scalable graphene production, flash graphene synthesis, pencil graphite precursors

## Abstract

The production of graphene from cost-effective and readily available sources remains a significant challenge in materials science. This study investigates the potential of common pencil leads as precursors for graphene synthesis using the Flash Joule Heating (FJH) process. We examined 6H, 4B, and 14B pencil grades, representing different graphite-to-clay ratios, under varying voltages (0 V, 200 V, and 400 V) to elucidate the relationships among initial composition, applied voltage, and resulting graphene quality. Samples were characterized using Raman spectroscopy, electrical resistance measurements, and microscopic analysis. The results revealed grade-specific responses to applied voltages, with all samples showing decreased electrical resistance post-FJH treatment. Raman spectroscopy indicated significant structural changes, particularly in I_D_/I_G_ and I_2D_/I_G_ ratios, providing insights into defect density and layer stacking. Notably, the 14B pencil lead exhibited unique behavior at 400 V, with a decrease in the I_D_/I_G_ ratio from 0.135 to 0.031 and an increase in crystallite size from 143 nm to 612 nm, suggesting potential in situ annealing effects. In contrast, harder grades (6H and 4B) showed increased defect density at higher voltages. This research contributes to the development of more efficient and environmentally friendly methods for graphene production, potentially opening new avenues for sustainable and scalable synthesis.

## 1. Introduction

Carbon, a fundamental element in nature, forms the basis of numerous materials, including graphite [1]. Among these, graphene—a two-dimensional (2D) material comprising a single layer of sp2-bonded carbon atoms arranged in a hexagonal lattice—has garnered significant attention because of its unique combination of electrical, mechanical, optical, thermal, and sensing properties. These exceptional characteristics have positioned graphene at the forefront of research across various technological fields [2,3,4,5,6].

Traditional methods for producing graphene, such as mechanical exfoliation, chemical reduction, and chemical vapor deposition (CVD), have been well-established [7,8]. However, these techniques often face challenges in terms of economic viability and environmental sustainability. Mechanical exfoliation, while producing high-quality graphene, is labor-intensive and yields limited quantities. Chemical reduction methods involve hazardous chemicals, raising environmental concerns. CVD, despite its ability to produce large-area graphene, requires high temperatures and costly equipment, limiting its scalability [9,10,11].

In response to these challenges, Flash Joule Heating (FJH) has emerged as a promising alternative for graphene synthesis [12,13]. This novel technique involves a rapid, high-temperature process capable of converting a wide range of carbon-containing materials into graphene [14,15]. FJH addresses not only the limitations of traditional methods but also the global challenge of managing over 2 billion tons of annual solid waste [16,17]. The process is characterized by its simplicity and operational ease, though it requires substantial energy input to achieve the necessary high temperatures instantaneously [18,19,20].

Recent studies have demonstrated the versatility of FJH in converting diverse waste materials into high-quality graphene. Researchers have successfully transformed plastic waste [21,22], domestic coal [23], and biomass waste [24,25] into graphene, showcasing significant environmental and energy consumption advantages over traditional synthesis techniques. Additionally, the integration of scientific machine learning frameworks has enhanced our understanding of the FJH process, leading to improvements in predicting graphene yield [26,27].

The quality and yield of graphene produced via FJH are significantly influenced by the carbon content and presence of impurities in the feedstock [28,29]. This variability necessitates tailored approaches to optimize graphene production for different precursor materials.

In this context, our study aims to investigate the potential of pencil leads as precursors for graphene synthesis via FJH. Pencil leads, being composites of graphite and clay, represent an abundant and low-cost source of carbon with high electrical conductivity and a layered structure, making them promising candidates for graphene production. However, the varying graphite-to-clay ratios in different types of pencil leads may affect the carbon content and impurity levels, potentially influencing the quality and yield of the synthesized graphene.

The primary objectives of this research are to investigate systematically the effects of different graphite-to-clay ratios in pencil leads on graphene synthesis via FJH, optimize FJH process parameters, particularly applied voltage, for maximizing graphene yield and quality from pencil lead precursors, and compare the structural and electrochemical properties of graphene samples synthesized under various conditions to identify the most effective synthesis method.

To achieve these objectives, we conducted a series of experiments varying the graphite-to-clay ratio in pencil leads and applying FJH at different voltages. The resulting graphene samples were thoroughly characterized to assess their quality and properties.

By elucidating the influence of pencil lead composition and processing conditions on the FJH process, this research aims to contribute to the development of more efficient and environmentally friendly methods for graphene production, potentially opening new avenues for sustainable and scalable graphene synthesis.

## 2. Materials and Methods

### 2.1. Preparation of Pencil Leads

**Figure 1:**
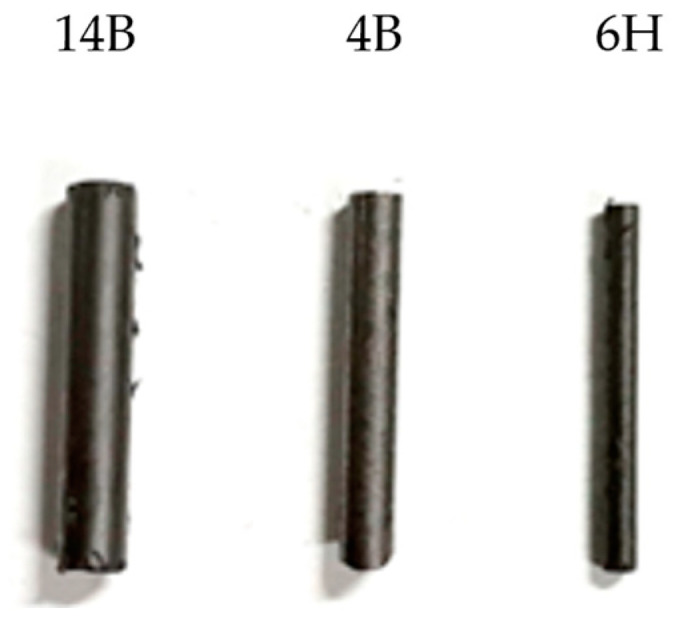
Various types of pencil grades used for the experiment.

Three distinct grades of pencil leads, i.e., 6H, 4B, and 14B, were carefully selected for this experiment, as shown in Figure 1. The 6H (lightest) lead, measuring 2 mm in diameter, contained minimal graphite content. The 4B (medium) lead, with a 3 mm diameter, had moderate graphite content, while the 14B (darkest) lead, 4 mm in diameter, possessed a rich graphite composition.

These pencil leads, sourced from a local supplier and manufactured by de Goya, were meticulously stripped of their wooden casings and cleaned to remove any residual wood particles.

To ensure experimental consistency, equal lengths of each pencil lead type were utilized. The graphite leads were precisely cut into 2 cm segments, providing consistent reactivity to high voltages and compatibility with the Flash Joule Heating (FJH) equipment.

Table 1 illustrates the composition of each pencil grade used in the experiments, detailing the percentages of graphite, clay, and wax [30]. Notably, the 6H pencil contains only 50% graphite, while the 14B is composed of more than 90% graphite, offering a wide range of carbon content for our study.

The selection of these specific pencil grades (6H, 4B, 14B) was deliberate, aiming to investigate the impact of varying graphite content on graphene synthesis via FJH. This range allowed us to examine how different carbon concentrations and impurity levels affect the quality and yield of the produced graphene.

For context, Table 2 presents the approximate percentages of carbon content in various waste materials commonly used in Flash Joule Heating processes for graphene production. This comparison highlights the potential advantages of using pencil leads as precursors, given their high and controllable carbon content.

The varying carbon content in these materials is relevant to our study as it influences the efficiency and quality of graphene production via FJH. By using pencil leads with known and varied graphite concentrations, we aim to optimize the FJH process for graphene synthesis and potentially provide insights applicable to a broader range of carbon-rich precursors.

### 2.2. Flash Joule Heating Setup

**Figure 2:**
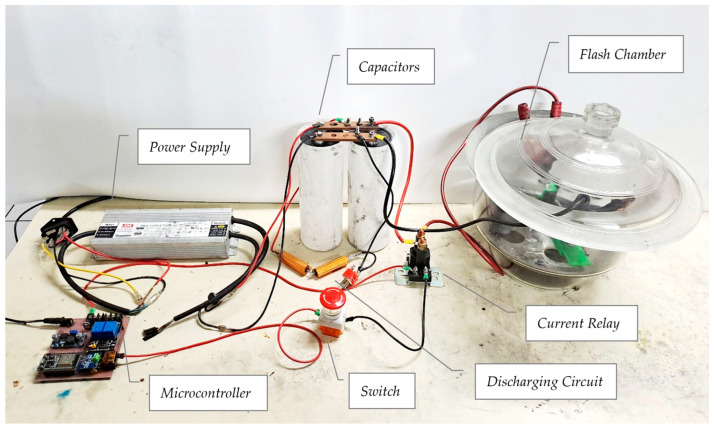
Experimental setup for Flash Joule Heating.

The Flash Joule Heating apparatus was designed to synthesize high-quality graphene from pencil lead precursors efficiently, addressing the need for a scalable, energy-efficient, and environmentally friendly graphene production method. The setup, as shown in Figure 2, comprises the following key components: a power supply (MeanWell HLG-320H-C700B LED driver, 220/230 V input, 435 V output), an ESP32 DEVKIT V1 microcontroller, a high-voltage capacitor (Kemet 6000 μF, 450 VDC), a 2-pin emergency stop switch, a high-current relay, a custom 3D-printed PLA sample holder, and a glass container.

The power supply provides the high voltage necessary for rapid Joule heating, chosen for its stable output and compatibility with the required voltage range. High-voltage capacitors store this output voltage and are connected in parallel via copper bus bars to increase power capacity, allowing for rapid energy discharge during the flash heating process.

**Figure 3:**
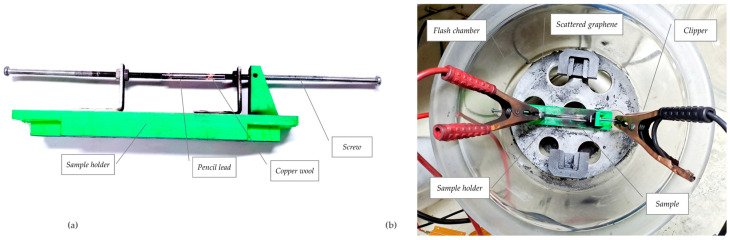
(**a**) Design of the sample holder structure fabricated with a 3D printer. (**b**) Internal view of the flash chamber.

The custom-designed, 3D-printed PLA sample holder houses the pencil lead precursor during the FJH process, as shown in Figure 3a. Its thermal resistance and adaptability make it ideal for this application. BOMEX glass pipettes serve as containment vessels within the holder. Copper wool electrodes ensure uniform current flow and seal gaps in the reaction chamber, offering excellent conductivity and conformability. The entire assembly is placed in the glass flash chamber for safety, as shown in Figure 3b, containing potential bursts at high voltages, smoke, and scattering of graphene during the reaction.

Safety features are integral to the design. A 2-pin emergency stop switch allows for immediate discharge of capacitors through the precursors, while a high-current relay enables controlled power delivery. Stripping tape is applied around glass tubes to prevent shattering during high-voltage operations.

The control and monitoring system consists of an ESP32 DEVKIT V1 microcontroller and a digital multimeter, which monitors capacitor voltage in real time, enabling precise control of the FJH process. Precision resistors (1% tolerance) are incorporated into the bleed resistor set to ensure accurate voltage settings during capacitor charging and discharging. Terminal blocks facilitate secure connections among the microcontroller, relay system, and other electrical components.

**Figure 4:**
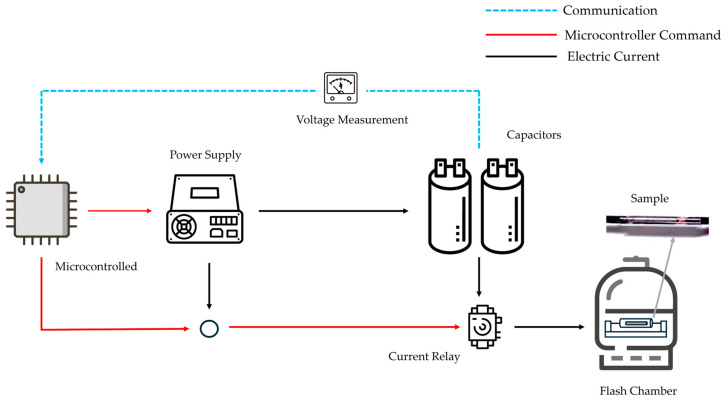
Flash Joule Heating setup block diagram.

This comprehensive setup enables the efficient conversion of pencil lead precursors into high-quality graphene-like material through controlled Flash Joule Heating. The design’s emphasis on precise control, safety, and analytical capabilities addresses the goals of scalability, energy efficiency, and environmental friendliness in graphene production. Figure 4 presents the block diagram that provides an overview of the entire Flash Joule Heating setup, illustrating the components and their interactions.

### 2.3. Post-Synthesis Analysis

For post-synthesis analysis, a Horiba Scientific LabRAM HR Raman spectrometer was utilized with specific parameters as follows: a 532 nm Edge laser, a hole setting of 499.984, a grating of 1800 (450–850 nm), and a 50% ND filter. The objective used was ×50 VIS LWD, and the analysis range spanned from 100 to 3300 cm^−1^. Each spectrum was acquired over 10 s with 10 accumulations.

To quantify the structural changes observed in the Raman spectra results, we employed the Tuinstra–Koenig relation [44],

La = (2.4 × 10^−10^) λ^4^ (I_D_/I_G_)^−1^ (1)

where λ is the laser wavelength (assuming 532 nm). This allowed us to estimate the crystallite size at each voltage. The conversion rates were estimated based on a combination of these parameters, reflecting the transformation from bulk graphite towards graphene-like structures. We developed the following formula to calculate these rates:

Conversion Rate (%) = [w_1_ × (1 − I_D_/I_G_) + w_2_ × (I_2D_/I_G_) + w_3_ × (1 − CS/CS_max_)] × 100% (2)

where the intensity ratios I_D_/I_G_ and I_2D_/I_G_ are used as key indicators of graphene quality and layer number. The crystalline size is considered as an additional factor, w_1_, w_2_, and w_3_ are weighting factors (where w_1_ = 0.4, w_2_ = 0.4, and w_3_ = 0.2), and CS_max_ is the maximum crystalline size observed (612 nm in this case). In this formula, a lower I_D_/I_G_ ratio indicates fewer defects, a higher I_2D_/I_G_ ratio suggests fewer layers [45], and a smaller crystalline size could indicate more exfoliation [46].

Moreover, electrical conductivity measurements were carried out using a Fluke-289 multimeter with a precision of 0.001 Ω to evaluate the resistance before and after the FJH process.

## 3. Results and Discussion

### 3.1. Physical Appearance Observations

**Figure 5:**
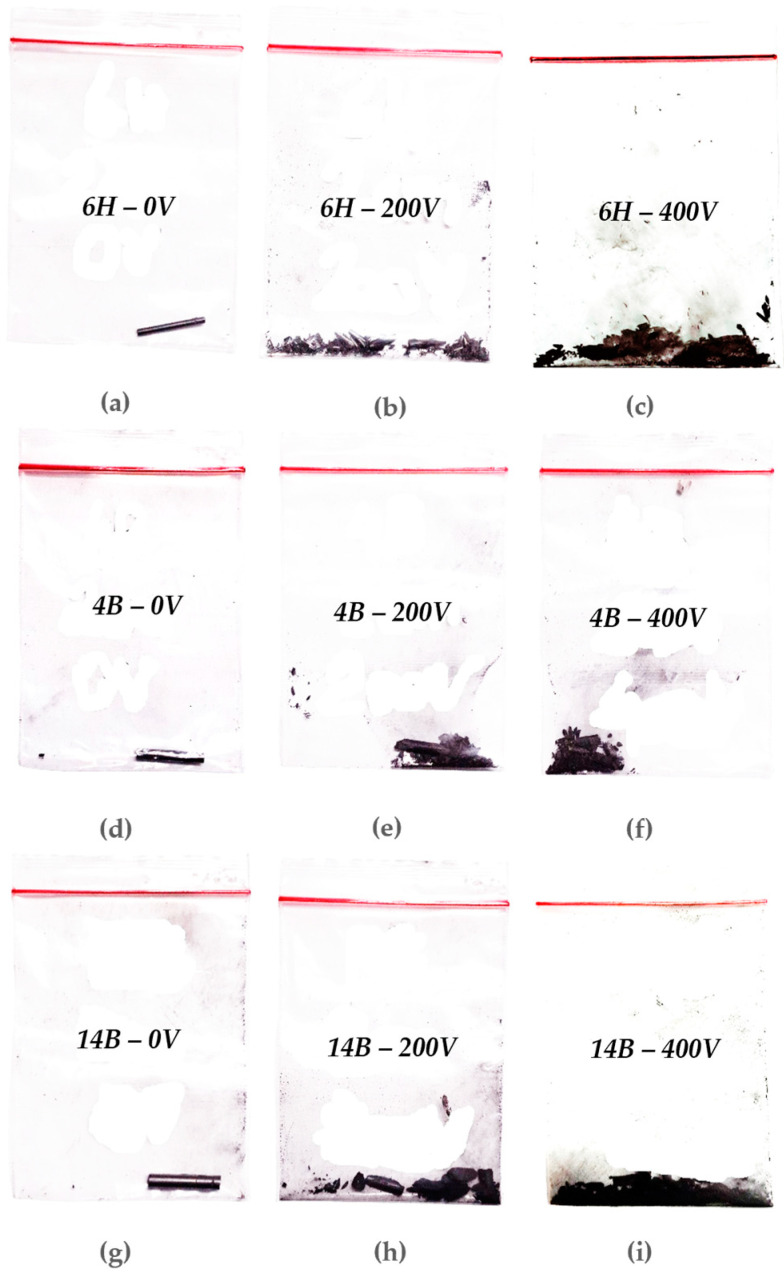
Changes in the physical appearance of the samples at different voltages: (**a**) 6H pencil at 0 V, (**b**) 6H pencil at 200 V, (**c**) 6H pencil at 400 V, (**d**) 4B pencil at 0 V, (**e**) 4B pencil at 200 V, (**f**) 4B pencil at 400 V, (**g**) 14B pencil at 0 V, (**h**) 14B pencil at 200 V, and (**i**) 14B pencil at 400 V.

The Flash Joule Heating (FJH) process induced distinctive physical changes in the graphite leads across different voltages, providing visual evidence of the transformation process. Figure 5 illustrates these changes for the 6H, 4B, and 14B pencil leads at 0 V, 200 V, and 400 V. At 100 V, all pencil grades exhibited minimal visible alterations. However, at 200 V, significant changes became apparent. The edges of the leads began to disintegrate into a powdery form, while the core showed signs of fracturing, indicating the onset of structural disorder. This transition was most pronounced in the softer 4B and 14B leads because of their higher graphite-to-clay ratio. Upon reaching 400 V, dramatic transformations occurred across all samples. The pencil leads almost entirely converted into an ash-like powdery substance, demonstrating the substantial energy input at this voltage level. This complete alteration suggests a threshold voltage for full conversion of solid pencil lead at this specific length.

The 6H pencil, despite its hardness, showed unexpected behavior. While resistant to breakage at high voltages, it paradoxically tended to burst at lower voltages. Notably, because of its higher impurity or clay ratio, the 6H lead turned into more crystalline-like particles compared with the 4B and 14B softer pencils. This inconsistency highlights the challenges in achieving uniform conversion for harder lead compositions. In contrast, the 4B pencil demonstrated the most consistent behavior across voltage levels. Its balanced graphite-to-clay ratio facilitated more predictable and manageable conversion rates, making it an ideal candidate for controlled graphene production. The 14B pencil, with its high graphite content and correspondingly low resistance, exhibited the most rapid and thorough conversion to ash-like powder at high voltages. However, this same quality made it prone to explosive reactions at maximum voltage levels. Notably, the 14B sample enabled complete capacitor discharge at full voltage, indicating its superior conductivity. These observations reveal the critical role of pencil lead composition in the FJH process. The varying behaviors of different grades underscore the need for careful selection of precursor materials to optimize graphene production efficiency and quality.

### 3.2. Resistance Measurement

The resistance values of the pencils before and after FJH treatment were measured at varying voltages, as shown in Table 3. Initially, we measured the initial resistance of each pencil type at 0 V. The 6H pencil exhibited the highest resistance, likely because of its lower graphite content. In contrast, the 4B and 14B pencils showed lower resistance, reflecting their higher graphite concentration. Notably, despite its larger diameter, the 14B pencil had higher resistance than 4B, suggesting that factors beyond graphite content influence electrical properties.

We then measured resistance after the FJH process at different voltages (200 V and 400 V). All pencil types showed a marked decrease in resistance with increasing applied voltage, indicating structural modifications that enhanced electrical conductivity. At 200 V, we observed a moderate drop in resistance across all samples, suggesting partial conversion of graphite into a more conductive material. At 400 V, the resistance decreased significantly for all pencil types, indicating a substantial enhancement in electrical conductivity. This likely results from a more complete conversion into a disordered, highly conductive structure.

The 6H pencil exhibited the most dramatic reduction in resistance, from 17.5 Ω at 0 V to 1.76 Ω at 400 V, a 90% decrease. This suggests that harder pencils, despite higher initial resistance, may undergo more extensive structural changes during FJH. The 4B and 14B pencils, while starting with lower resistance, showed proportionally smaller reductions. This could indicate that softer pencils with higher initial graphite content may require less structural modification to achieve high conductivity.

These resistance measurements provide quantitative evidence of the FJH process’s effectiveness in transforming pencil lead into more conductive materials, likely graphene or related carbon nanostructures. The varying responses among the pencil types underscore the importance of precursor composition in optimizing the FJH process for graphene production.

### 3.3. Performance Analysis of Pencils

To understand the behavior of different pencil grades in the Flash Joule Heating (FJH) process, we conducted simulations of capacitor discharge through 4B, 14B, and 6H pencil leads.

**Figure 6:**
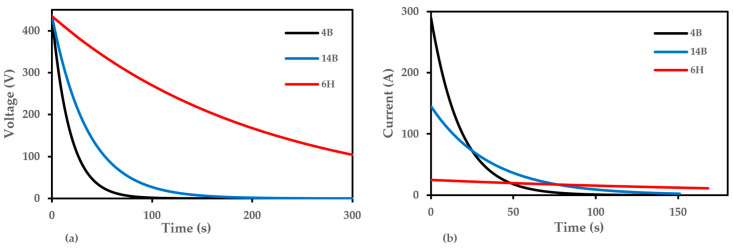
Capacitor discharge (**a**) voltage concerning time and (**b**) current concerning time decay through each pencil grade during the FJH process.

Figure 6 illustrates the voltage and current dynamics during this process. Figure 6a shows the voltage decay across the flash chamber for each pencil grade. The 6H pencil, with the highest resistance of 17.5 ohms, exhibits the slowest voltage drop, maintaining a higher voltage for an extended period. This high resistance may result in less efficient energy transfer during the FJH process. The 14B pencil, with a moderate resistance of 3 ohms, shows an intermediate rate of voltage decay. In contrast, the 4B pencil, having the lowest resistance of 1.5 ohms, demonstrates the fastest voltage drop, reaching 0 V more quickly than the others. This rapid decay suggests more efficient energy dissipation, which could be beneficial for graphene production.

Figure 6b illustrates the current flow through each pencil grade during discharge. The 4B pencil, with its low resistance, exhibits the highest initial current spike, exceeding 300 A, followed by a rapid decay. This high current capacity suggests it may be the most efficient for the FJH process. The 14B pencil shows a moderate current peak around 150 A, with a slower decay compared with 4B, consistent with its intermediate resistance. The 6H pencil, because of its high resistance, maintains a consistently low current, near 0 A, throughout the discharge process, indicating its unsuitability for efficient FJH.

**Figure 7:**
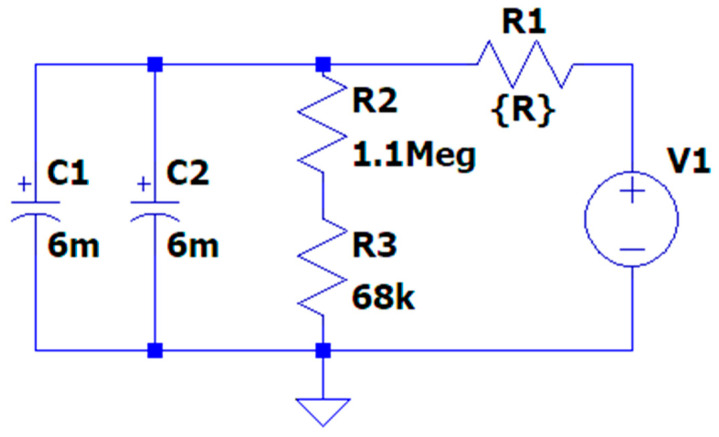
Simplified circuit diagram of the Flash Joule Heating (FJH) setup used in the simulation.

These results suggest that pencils with lower resistance, such as 4B and 14B, are more suitable for graphene production via FJH. Their ability to allow higher current flow and rapid energy dissipation likely contributes to more efficient heating and graphene formation. To better understand these results, we modeled the FJH setup using the circuit shown in Figure 7. This simplified circuit includes two 6 mF or 6000 μF capacitors (C1 and C2) representing the capacitor bank, fixed resistors R2 (1.1 MΩ) and R3 (68 kΩ), and a variable resistor R1 representing the pencil lead. The resistance values for R1 were set to 1.5 ohms for 4B, 3 ohms for 14B, and 17.5 ohms for 6H, based on our measurements of the actual pencil leads used in the experiments. This simulation provides valuable insights into the electrical behavior of different pencil grades under FJH conditions, helping to predict their effectiveness in the graphene production process.

### 3.4. Raman Spectroscopy and Microscopic Analysis

Raman spectroscopy has emerged as a powerful, non-destructive tool for characterizing carbon-based materials, particularly in the study of graphene and its derivatives. This technique provides crucial insights into the structural and electronic properties of these materials through the analysis of characteristic spectral features. In graphene-related structures, the Raman spectrum typically exhibits the following three primary bands: the D-band (~1350 cm^−1^), associated with defects and disorder, the G-band (~1580 cm^−1^), representing in-plane vibrations of sp^2^ carbon atoms, and the 2D-band (~2700 cm^−1^), an overtone of the D-band that is particularly sensitive to the number of graphene layers [47,48].

The intensity ratios of these bands, specifically I_D_/I_G_ and I_2D_/I_G_, offer valuable information about the quality and structure of graphene-like materials. The I_D_/I_G_ ratio serves as an indicator of defect density, with lower values suggesting fewer defects and higher structural order. Conversely, the I_2D_/I_G_ ratio provides insights into the number of graphene layers, with higher values typically associated with fewer layers [49]. Additionally, the positions and shapes of these bands can reveal information about doping, strain, and other structural characteristics [50].

Recent advancements in Raman spectroscopy have further enhanced its capabilities in graphene research. For instance, tip-enhanced Raman spectroscopy (TERS) now allows for nanoscale spatial resolution, enabling the study of local defects and edge states in graphene [51]. Furthermore, the development of in situ Raman techniques has facilitated real-time monitoring of graphene formation and modification processes, providing unprecedented insights into the dynamics of these transformations [52].

#### 3.4.1. Detail Analysis of the 6H Pencil

**Figure 8:**
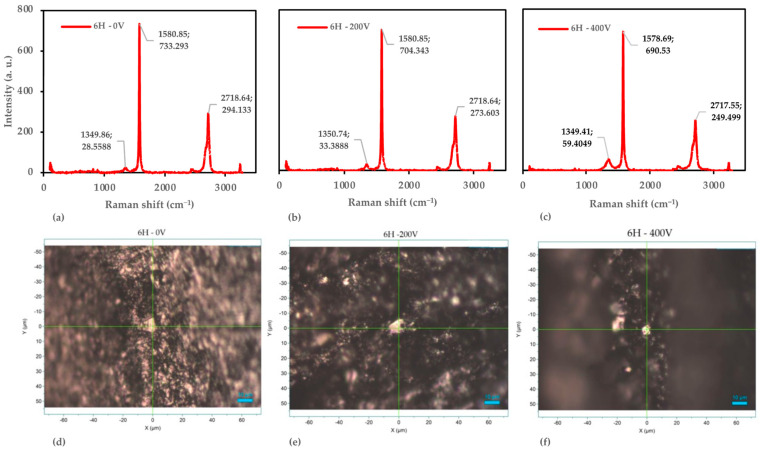
Raman spectroscopy and optical microscopy analysis of the 6H pencil samples. (**a**) Raman spectra at 0 V. (**b**) Raman spectra at 200 V. (**c**) Raman spectra at 400 V. (**d**) Optical microscopy image at 0 V. (**e**) Optical microscopy image at 200 V. (**f**) Optical microscopy image at 400 V.

In our study, we applied Raman spectroscopy to investigate the structural changes in the 6H pencil lead samples subjected to different voltages (0 V, 200 V, and 400 V) as shown in Figure 8. The results reveal a fascinating progression from a well-ordered graphitic structure towards few-layer or multi-layer graphene-like materials.

At 0 V, the sample exhibits characteristics typical of a well-ordered graphitic structure. The G-band appears at 1580.85 cm^−1^ with an intensity of 733.293 counts, while the D-band is observed at 1249.86 cm^−1^ (28.5588 counts) and the 2D-band at 2718.64 cm^−1^ (294.133 counts). The low I_D_/I_G_ ratio of 0.039 indicates minimal defects, comparable to high-quality graphite [45]. The I_2D_/I_G_ ratio of 0.401 suggests a multi-layer graphitic structure, consistent with bulk graphite [53]. Interestingly, the D-band position (1249.86 cm^−1^) is lower than typical graphite (~1350 cm^−1^), which may be attributed to the clay content in pencil lead, as observed by Németh et al. in their study of graphite–clay composites [54].

As the voltage increases to 200 V, notable changes occur in the Raman spectrum. While the G-band remains at 1580.85 cm^−1^ (704.343 counts), the D-band shifts to 1350.74 cm^−1^ (33.3888 counts), aligning more closely with typical graphene-like structures [55]. The 2D-band is observed at 2718.64 cm^−1^ (273.603 counts). The slight increase in the I_D_/I_G_ ratio to 0.047 suggests the introduction of defects, potentially indicating the onset of graphene formation. This observation is consistent with the findings of Paton et al., who reported I_D_/I_G_ ratios of 0.04–0.3 for liquid-phase exfoliated graphene [56].

At 400 V, further structural changes become evident. The G-band slightly shifts to 1578.69 cm^−1^ (690.53 counts), while the D-band appears at 1349.41 cm^−1^ (59.4049 counts) and the 2D-band at 2717.55 cm^−1^ (249.499 counts). The increased I_D_/I_G_ ratio of 0.086 indicates a significant rise in defects and disorder, consistent with the formation of graphene-like structures. This value falls within the range reported by Cançado et al. for nano-graphite (0.02–0.3) [57]. The slight G-band shift to lower wavenumbers could suggest strain in the graphene layers or a reduction in the number of layers, as observed by Lee et al. in their study of few-layer graphene [58].

To quantify these changes, we employed the Tuinstra–Koenig relation [44], this allowed us to estimate the crystallite size at each voltage as follows: 493 nm at 0 V, 406 nm at 200 V, and 223 nm at 400 V. This decrease in crystallite size corroborates the increasing disorder with applied voltage, aligning with the observations of Cançado et al. for the evolution of graphite to nanocrystalline graphite [57].

Complementing the Raman analysis, the microscopic examination reveals progressive morphological changes in the samples. At 0 V, we observe a relatively uniform surface with minor particulate features. As the voltage increases to 200 V, more pronounced features and increased particle clustering become evident. At 400 V, significant morphological changes occur, including higher levels of aggregation and structural disorder. These observations are consistent with the findings reported in studies on the thermal reduction of graphene oxide (GO) paper, where increasing reduction temperatures resulted in pronounced changes in surface morphology, including the suppression of wrinkles and the emergence of granular structures [59].

The combined Raman and microscopic analysis demonstrate that applying voltage to the 6H samples induces structural changes, increasing defects and disorder. This progression is consistent with the transformation of graphite into few-layer or multi-layer graphene structures. However, the I_2D_/I_G_ ratios remain lower than typically reported for high-quality monolayer graphene (~2–4) [60], suggesting our samples likely consist of few-layer or multi-layer graphene structures.

These results align with our earlier resistance measurements, which showed decreasing resistance with increasing voltage, and the physical appearance observations of increased powdery texture at higher voltages. The findings suggest that voltage application to pencil lead could be a promising method for producing graphene-like materials, although further optimization may be required to achieve high-quality monolayer graphene.

#### 3.4.2. Detail Analysis of the 4B Pencil

**Figure 9:**
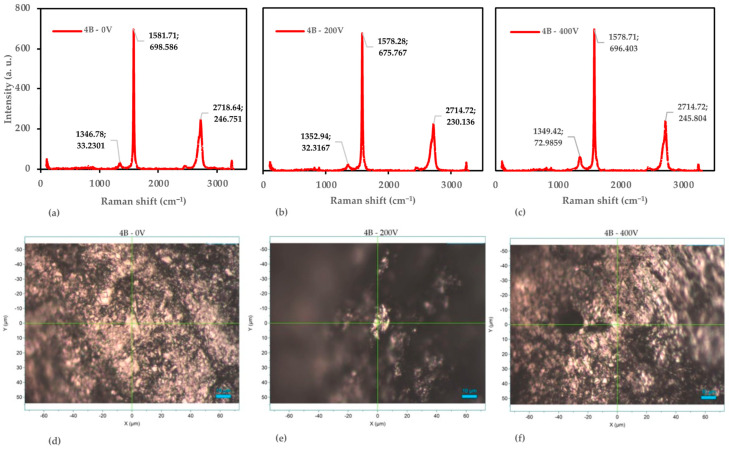
Raman spectroscopy and optical microscopy analysis of the 4B pencil samples. (**a**) Raman spectra at 0 V. (**b**) Raman spectra at 200 V. (**c**) Raman spectra at 400 V. (**d**) Optical microscopy image at 0 V. (**e**) Optical microscopy image at 200 V. (**f**) Optical microscopy image at 400 V.

Following our examination of the 6H pencil lead, we extended our investigation to the 4B pencil samples, subjecting them to the same voltage conditions (0 V, 200 V, and 400 V). Figure 9 presents the combined Raman spectroscopy and microscopic analysis for these 4B samples, revealing intriguing structural changes as voltage increases.

At 0 V, the 4B sample exhibits characteristics of a well-ordered graphitic structure, albeit with some notable differences from the 6H sample. The G-band appears at 1581.71 cm^−1^ with an intensity of 698.586, while the D-band is observed at 1346.78 cm^−1^ (33.2301 counts) and the 2D-band at 2718.64 cm^−1^ (246.751 counts). The I_D_/I_G_ ratio of 0.048 indicates a relatively low defect density, though slightly higher than the 6H sample at 0 V. This suggests that the 4B pencil lead inherently contains more structural disorder, which is consistent with its softer nature [45]. The I_2D_/I_G_ ratio of 0.353 further supports a multi-layer graphitic structure, typical of bulk graphite [53].

Interestingly, the D-band position (1346.78 cm^−1^) in the 4B sample is closer to the typical graphite value (~1350 cm^−1^) compared with the 6H sample. This could indicate a difference in the clay content or composition between the two pencil grades, affecting their Raman signatures [54]. The microscopic image for the 4B–0 V sample reveals a surface with minor particulate features, consistent with the relatively low I_D_/I_G_ ratio and suggesting a fairly uniform morphology.

As we increase the voltage to 200 V, subtle yet noteworthy changes occur in the Raman spectrum. The G-band shifts slightly to 1578.28 cm^−1^ (675.767 counts), the D-band moves to 1352.94 cm^−1^ (32.3167 counts), and the 2D-band shifts to 2714.72 cm^−1^ (230.136 counts). Remarkably, the I_D_/I_G_ ratio remains stable at 0.048, suggesting that the defect density is maintained at this voltage level. This behavior differs from the 6H sample, which shows an increase in defects at 200 V. The I_2D_/I_G_ ratio slightly decreases to 0.341, indicating minor changes in the graphitic layer stacking [55]. The microscopic image for the 4B–200 V sample shows fewer pronounced features and less particle clustering compared with 0 V, reflecting a consistent surface morphology with only minor alterations.

At 400 V, we observe more significant structural changes. The G-band shifts to 1579.71 cm^−1^ (696.403 counts), the D-band to 1349.42 cm^−1^ (72.9859 counts), and the 2D-band remains at 2714.72 cm^−1^ (245.804 counts). The I_D_/I_G_ ratio increases substantially to 0.105, indicating a significant rise in defects and disorder within the structure. This increase is more pronounced than in the 6H sample at the same voltage, suggesting that the 4B pencil lead is more susceptible to voltage-induced structural changes [56]. Interestingly, the I_2D_/I_G_ ratio remains relatively stable at 0.353, reflecting consistent structural integrity of the graphitic layers despite the increased defect density.

The microscopic image for the 4B–400 V sample reveals significant morphological changes, including higher levels of aggregation and structural reorganization. This visual evidence corroborates the Raman data, indicating the introduction of defects at higher voltages and resulting in a more irregular surface morphology.

To quantify these structural changes, we again employed the Tuinstra–Koenig relation [44]. The estimated crystallite sizes for the 4B samples are 405 nm at 0 V, 402 nm at 200 V, and 183 nm at 400 V. This dramatic decrease in crystallite size at 400 V aligns with the significant increase in the I_D_/I_G_ ratio and the observed morphological changes.

The analysis of the 4B pencil lead demonstrates that applying voltage induces structural changes, primarily by increasing defects while maintaining overall graphitic layer integrity. This behavior differs somewhat from the 6H samples, particularly in the stability of the defect density at lower voltages and the more pronounced increase in defects at higher voltages. These differences likely stem from the inherent structural and compositional variations between the 4B and 6H pencil leads [57].

These findings contribute to our understanding of how different grades of pencil lead respond to electrical stress, potentially offering insights into optimizing the production of graphene-like materials from readily available sources. These results also highlight the importance of considering the initial graphite quality and composition when developing voltage-based exfoliation methods for graphene production [56].

#### 3.4.3. Detail Analysis of the 14B Pencil

**Figure 10:**
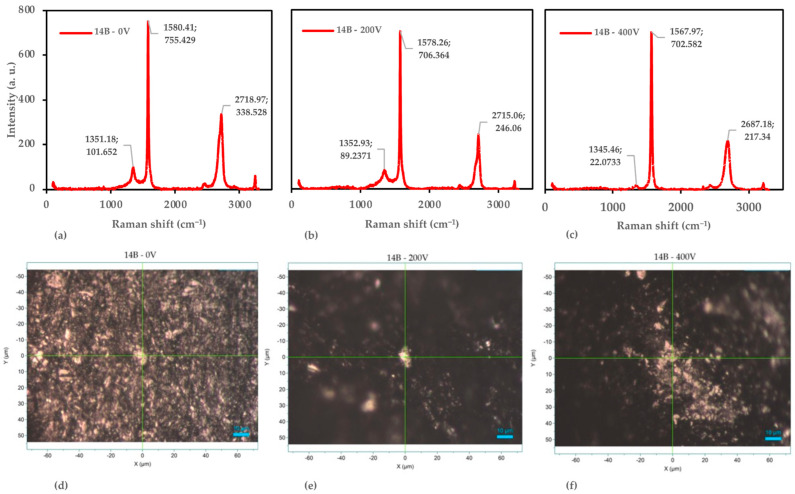
Raman spectroscopy and optical microscopy analysis of the 14B pencil samples. (**a**) Raman spectra at 0 V. (**b**) Raman spectra at 200 V. (**c**) Raman spectra at 400 V. (**d**) Optical microscopy image at 0 V. (**e**) Optical microscopy image at 200 V. (**f**) Optical microscopy image at 400 V.

Building upon our analyses of 6H and 4B pencil leads, we now turn our attention to the 14B samples, which represent the softest grade in our study. Figure 10 presents the combined Raman spectroscopy and microscopic analysis for 14B samples subjected to voltages of 0 V, 200 V, and 400 V, revealing intriguing structural changes that differ significantly from the harder pencil grades.

At 0 V, the 14B sample exhibits characteristics that reflect its softer nature and higher graphite content. The G-band appears at 1580.41 cm^−1^ with an intensity of 755.429 counts, while the D-band is observed at 1351.18 cm^−1^ (101.652 counts) and the 2D-band at 2718.97 cm^−1^ (338.528 counts). The I_D_/I_G_ ratio of 0.135 indicates a moderate defect density, significantly higher than both the 6H and 4B samples at 0 V. This aligns with expectations for softer pencil leads, which typically contain more graphite and fewer binders, resulting in a less ordered structure [45]. The I_2D_/I_G_ ratio of 0.448 suggests a multi-layer graphitic structure, but with potentially fewer layers or more exfoliated regions compared with the harder grades [53].

The D-band position (1351.18 cm^−1^) in the 14B sample closely matches the typical graphite value, indicating a composition dominated by graphite with minimal clay content [54]. The microscopic image for the 14B–0 V sample reveals a surface with considerable particulate features, consistent with the moderate I_D_/I_G_ ratio and reflecting the softer, more easily deformable nature of 14B lead.

As we increase the voltage to 200 V, we observe subtle yet intriguing changes in the Raman spectrum. The G-band shifts slightly to 1578.26 cm^−1^ (706.364 counts), the D-band moves to 1352.93 cm^−1^ (89.2371 counts), and the 2D-band shifts to 2715.06 cm^−1^ (246.06 counts). Notably, the I_D_/I_G_ ratio slightly decreases to 0.126, suggesting a minor reduction in structural defects. This behavior contrasts with the 6H and 4B samples, which showed either stability or increases in defect density at 200 V. The I_2D_/I_G_ ratio decreases to 0.348, indicating subtle changes in the graphitic layer stacking [55]. The microscopic image for the 14B–200 V sample shows fewer pronounced features and less particle clustering compared with 0 V, correlating with the slight reduction in I_D_/I_G_ and suggesting a smoothing or reorganization effect.

At 400 V, we observe dramatic structural changes that set the 14B sample apart from the harder grades. The G-band shifts significantly to 1567.97 cm^−1^ (702.582 counts), the D-band moves to 1345.46 cm^−1^ with a notably decreased intensity (22.0733 counts), and the 2D-band shifts to 2687.18 cm^−1^ (217.34 counts). Remarkably, the I_D_/I_G_ ratio decreases substantially to 0.031, indicating a significant reduction in defects and disorder within the structure. This is in stark contrast to the 6H and 4B samples, which show increased defect density at 400 V. The I_2D_/I_G_ ratio continues to decrease to 0.309, reflecting further structural changes.

The microscopic image for the 14B–400 V sample reveals significant morphological changes, including higher levels of aggregation and structural reorganization. This visual evidence, combined with the decreased I_D_/I_G_ ratio, suggests possible annealing or restructuring effects at higher voltages. Such behavior is reminiscent of thermal annealing processes in graphene materials, where high temperatures can lead to defect healing and structural reorganization [59].

To quantify these structural changes, we again employed the Tuinstra–Koenig relation [56]. The estimated crystallite sizes for the 14B samples are 143 nm at 0 V, 152 nm at 200 V, and 612 nm at 400 V. This dramatic increase in crystallite size at 400 V is consistent with the significant decrease in the I_D_/I_G_ ratio and supports the hypothesis of voltage-induced annealing or restructuring.

The analysis of the 14B pencil lead demonstrates a unique response to applied voltage, characterized by a reduction in defects and disorder, possibly accompanied by material restructuring. This behavior differs markedly from the harder pencil grades and may be attributed to the higher graphite content and lower binder concentration in the 14B lead [57]. The voltage-induced changes observed in the 14B samples suggest a potential route for producing higher-quality graphene-like materials from softer pencil leads, possibly through a combination of exfoliation and in situ annealing effects [56].

These findings highlight the complex interplay among initial graphite quality, composition, and voltage-induced structural changes in pencil lead materials. The unique behavior of the 14B samples opens new possibilities for tailoring voltage-based exfoliation methods to produce graphene materials with specific structural characteristics.

#### 3.4.4. Comparative Analysis of the 6H, 4B, and 14B Pencil Leads

**Figure 11:**
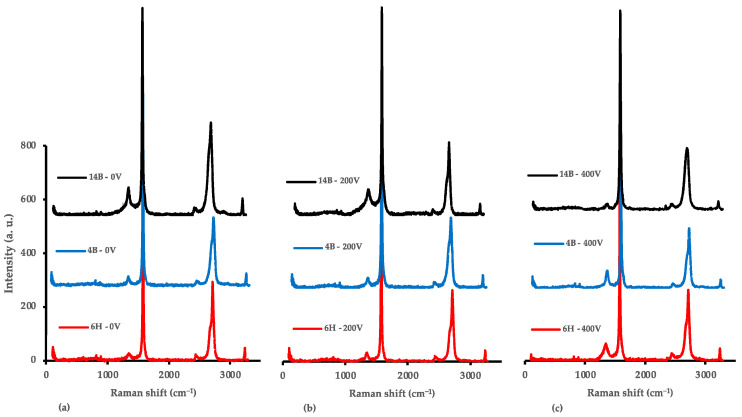
Raman spectroscopy comparison of the 6H, 4B, and 14B pencil samples at 0 V, 200 V, and 400 V. (**a**) Raman spectra comparison at 0 V. (**b**) Raman spectra comparison at 200 V. (**c**) Raman spectra comparison at 400 V.

The comparative analysis of the Raman spectroscopy results for the 6H, 4B, and 14B pencil leads across applied voltages of 0 V, 200 V, and 400 V reveals intriguing insights into the structural evolution of graphitic materials under electrical stress. Figure 11 presents a comprehensive side-by-side visualization of the Raman spectra, allowing for a direct comparison of voltage-induced changes across different pencil grades.

At 0 V, distinct differences in the initial structures of the three pencil grades are evident. The 6H sample exhibits the lowest I_D_/I_G_ ratio (0.039), indicating minimal initial defects, followed by 4B (0.048) and then 14B (0.135). This trend aligns with the expected graphite content and hardness of these pencils, where softer leads contain more graphite and typically exhibit greater structural disorder. The I_2D_/I_G_ ratios follow a similar pattern, with 14B showing the highest value (0.448), suggesting a more exfoliated or less tightly stacked graphitic structure compared with the harder grades. These initial structural differences play a crucial role in determining how each pencil grade responds to applied voltage.

Upon applying 200 V, each pencil grade demonstrates a unique response. The 6H sample shows a slight increase in I_D_/I_G_ (0.047), indicating a minor increase in defects. In contrast, the 4B sample maintains a stable I_D_/I_G_ ratio (0.048), suggesting resistance to defect formation at this voltage. Most intriguingly, the 14B sample exhibits a decrease in I_D_/I_G_ (0.126), hinting at a possible structural reorganization or defect-healing process. These varied responses underscore the importance of initial composition and structure in determining how pencil lead materials react to electrical stress, highlighting the complex interplay between material properties and external stimuli.

The most striking differences emerge at 400 V, where the behavior of the pencil grades diverges significantly. The 6H sample experiences a substantial increase in I_D_/I_G_ (0.086), indicating considerable defect formation. The 4B sample shows an even more pronounced increase in I_D_/I_G_ (0.105), suggesting greater susceptibility to voltage-induced defects. Remarkably, the 14B sample exhibits a dramatic decrease in I_D_/I_G_ (0.031), implying significant structural improvement or reorganization. This divergent behavior at high voltage is particularly intriguing, as it suggests that while harder grades (6H and 4B) undergo increased disorder, possibly because of the breakdown of their more rigid structures, the softer 14B grade appears to experience a process akin to voltage-induced annealing.

The evolution of the G-band and 2D-band positions provides further insights into the structural changes occurring within the samples. All grades show a general trend where the G-band shifts to lower wavenumbers with increasing voltage, which could indicate strain development or changes in layer interactions. The 2D-band evolution varies among the grades, with 14B showing the most significant changes, particularly at 400 V, where it shifts to a much lower wavenumber (2687.18 cm^−1^) compared with the other grades. These spectral shifts offer valuable information about the nature of the structural modifications induced by voltage application.

The analysis of crystallite size changes, calculated using the Tuinstra–Koenig relation, further emphasizes the unique behavior of different pencil grades. The 6H and 4B samples show a trend of decreasing crystallite size with increasing voltage, with 6H decreasing from 493 nm (0 V) to 223 nm (400 V), and 4B from 405 nm (0 V) to 183 nm (400 V). In stark contrast, the 14B sample exhibits an extraordinary increase in crystallite size, from 143 nm (0 V) to 612 nm (400 V). This dramatic growth in crystallite size for 14B at high voltage corroborates the observed decrease in the I_D_/I_G_ ratio and suggests a significant restructuring of the graphitic material.

The diverse responses of different pencil grades to applied voltage have important implications for potential applications and future research directions. The controlled defect introduction observed in harder pencil leads (6H, 4B) at lower voltages may be advantageous for applications requiring specific levels of structural disorder or exfoliation. Conversely, the apparent ability of softer pencil leads (14B) to undergo structural improvement at higher voltages opens exciting possibilities for high-quality graphene production. The voltage-induced changes observed, particularly in 14B, suggest opportunities for in situ modification of graphitic structures, potentially allowing for tailored material properties to suit specific applications.

Table 4 presents the Raman spectroscopy data and estimated conversion rates for the three pencil grades (6H, 4B, and 14B) subjected to different voltages (0 V, 200 V, and 400 V). The key parameters analyzed are the I_D_/I_G_ ratio (indicative of defects), the I_2D_/I_G_ ratio (related to layer thickness), and crystalline size. The conversion rates are estimated based on a combination of these parameters, reflecting the transformation from bulk graphite towards graphene-like structures. The percentages are based on a combination of factors, such as changes in the I_D_/I_G_ ratio, the I_2D_/I_G_ ratio, and crystallite size, as shown in Equation (2).

The 6H pencil lead demonstrated a low conversion, approximately 9% at 400 V. This grade showed a gradual increase in defects (I_D_/I_G_ ratio from 0.039 to 0.086) and a decrease in crystalline size (493 nm to 223 nm), indicating a controlled exfoliation process. The 4B grade exhibited a similar transformation, achieving an 8% conversion rate at 400 V. The significant increase in the I_D_/I_G_ ratio (0.047 to 0.105) and decrease in crystalline size (405 nm to 183 nm) at 400 V indicate substantial exfoliation and defect introduction. Remarkably, the 14B pencil lead showed the most dramatic transformation, reaching a 24% conversion rate at 400 V. This grade exhibited unique behavior, with a decrease in the I_D_/I_G_ ratio (0.135 to 0.031) and a substantial increase in crystalline size (143 nm to 612 nm) at higher voltages.

This comparative analysis underscores the complex relationship among initial graphite quality, composition, and voltage-induced structural changes in pencil lead materials. It highlights the potential for developing grade-specific and voltage-optimized processes for graphene production from readily available pencil lead sources. The findings suggest that by carefully selecting pencil grades and optimizing applied voltages, it may be possible to achieve precise control over the resulting graphene-like material properties. Additionally, this research emphasizes the importance of raw material purity in graphene synthesis via flash joule heating, as higher levels of impurities can lead to increased disorder in the resulting graphene structure. This insight underscores the need for refining waste materials before their use in graphene production. This avenue of research offers promising opportunities for advancing the field of graphene-based materials, potentially leading to more accessible and tailored production methods for a wide range of applications in electronics, energy storage, and beyond.

## 4. Conclusions

Our study reveals significant voltage-induced structural changes in pencil lead materials, with varying responses observed across different grades. The results show grade-specific responses to applied voltages, with all samples showing decreased electrical resistance after FJH treatment. Raman spectroscopy indicated significant structural changes, particularly in the I_D_/I_G_ and I_2D_/I_G_ ratios, providing insights into defect density and layer stacking. Particularly, the 14B pencil lead exhibited unique behavior at 400 V, with a decrease in the I_D_/I_G_ ratio from 0.135 to 0.031 and an increase in crystallite size from 143 nm to 612 nm, suggesting potential in situ annealing effects. On the other hand, the harder grades (6H and 4B) showed increased defect density at higher voltages. The exceptionally high conversion rate and improved structural characteristics observed in the 14B pencil lead at 400 V indicate that more graphite-rich leads may be particularly promising for the voltage-based production of high-quality graphene-like materials. These findings open new avenues for the development of simple, cost-effective methods for producing graphene-like materials from readily available sources. Further research is warranted to optimize voltage application parameters and explore the potential of other graphite-based materials for similar conversions.

## Figures and Tables

**Table 1:** Percentage value of graphite, clay, and wax particles for the utilized pencil grades.

Pencil Type	Graphite	Clay	Wax
6H	0.50	0.45	0.05
4B	0.79	0.15	0.05
14B	>0.90	<0.04	0.05

**Table 2:** Approximate percentage of carbon content in different waste materials.

Material	Carbon Content (%)	References
Biomass	45–50%	[31,32]
Municipal solid waste	20–30%	[33]
Plastic waste	60–80%	[34,35]
Paper/cardboard	40–50%	[36,37]
Food waste	30–50%	[38,39]
Wood	50–55%	[40,41]
Textile waste	35–50%	[42,43]

**Table 3:** Resistance values of pencils before and after FJH treatment at varying voltages.

Pencil Type	Resistance (0 V)	Resistance (200 V)	Resistance (400 V)
6H	17.5 Ω	4.76 Ω	1.76 Ω
4B	1.5 Ω	1.16 Ω	0.9 Ω
14B	3 Ω	2.3 Ω	1.25 Ω

**Table 4:** Comparative analysis of the Raman spectroscopy data and estimated conversion rates at varying voltages.

Pencil Grade	Voltage (V)	I_D_/I_G_	I_2D_/I_G_	Crystalline Size (nm)	Conversion Rate
6H	0	0.039	0.401	493	0% (baseline)
	200	0.047	0.389	406	4%
	400	0.086	0.361	223	9%
4B	0	0.047	0.353	405	0% (baseline)
	200	0.047	0.341	402	0.7%
	400	0.105	0.353	183	8%
14B	0	0.135	0.448	143	0% (baseline)
	200	0.126	0.348	152	5%
	400	0.031	0.309	612	24%

## Data Availability

Data are contained within the article.

## References

[1] Manocha L.M. (2022). Carbon Based Materials. Encyclopedia of Materials: Metals and Alloys.

[2] Asghar S., Al-Qoyyim T.M., Diarta M.H., Doyan A. (2023). Graphene: The Revolutionary 2D Material. J. Penelit. Pendidik. IPA.

[3] Urade A.R., Lahiri I., Suresh K.S. (2023). Graphene Properties, Synthesis and Applications: A Review. JOM.

[4] Xu S., Wang T., Liu G., Cao Z., Frank L.A., Jiang S., Zhang C., Li Z., Krasitskaya V.V., Li Q. (2021). Analysis of interactions between proteins and small-molecule drugs by a biosensor based on a graphene field-effect transistor. Sens. Actuators B Chem..

[5] Xu S., Zhang C., Jiang S., Hu G., Li X., Zou Y., Liu H., Li J., Li Z., Wang X. (2019). Graphene foam field-effect transistor for ultra-sensitive label-free detection of ATP. Sens. Actuators B Chem..

[6] Tian M., Qiao M., Shen C., Meng F., Frank L.A., Krasitskaya V.V., Wang T., Zhang X., Song R., Li Y. (2020). Highly-sensitive graphene field effect transistor biosensor using PNA and DNA probes for RNA detection. Appl. Surf. Sci..

[7] Bhuyan M.S.A., Uddin M.N., Islam M., Bipasha F.A., Hossain S.S. (2016). Synthesis of graphene. Int. Nano Lett..

[8] Yip T.M., Tong G.B. (2023). Fabrication Routes of Graphene. Graphene.

[9] Vinci G., Gobbi L., Ruggieri R., Ruggeri M., Tiradritti M. (2023). Challenges and opportunities for the graphene industry. Sustainable approaches for the circular economy. Graphene Extraction from Waste.

[10] Al Faruque A., Syduzzaman, Sarkar J., Bilisik K., Naebe M. (2021). A Review on the Production Methods and Applications of Graphene-Based Materials. Nanomaterials.

[11] Santhiran A., Iyngaran P., Abiman P., Kuganathan N. (2021). Graphene Synthesis and Its Recent Advances in Applications—A Review. C.

[12] Wyss K.M., Luong D.X., Tour J.M. (2022). Large-Scale Syntheses of 2D Materials: Flash Joule Heating and Other Methods. Adv. Mater..

[13] Sun Z., Hu Y.H. (2020). Ultrafast, Low-Cost, and Mass Production of High-Quality Graphene. Angew. Chem. Int. Ed..

[14] Luong D.X., Bets K.V., Algozeeb W.A., Stanford M.G., Kittrell C., Chen W., Salvatierra R.V., Ren M., McHugh E.A., Advincula P.A. (2020). Gram-scale bottom-up flash graphene synthesis. Nature.

[15] Deng B., Luong D.X., Wang Z., Kittrell C., McHugh E.A., Tour J.M. (2021). Urban mining by flash Joule heating. Nat. Commun..

[16] Wyss K.M., Deng B., Tour J.M. (2023). Upcycling and urban mining for nanomaterial synthesis. Nano Today.

[17] Barbhuiya N.H., Kumar A., Singh A., Chandel M.K., Arnusch C.J., Tour J.M., Singh S.P. (2021). The Future of Flash Graphene for the Sustainable Management of Solid Waste. ACS Nano.

[18] Liao Y., Zhu R., Zhang W., Liu Z., Zhu H., Sun Y. (2023). Ultrafast synthesis of novel coal-based graphene and its anticorrosion properties of epoxy/graphene nanocomposite coatings. Prog. Org. Coat..

[19] Liu X., Luo H. (2024). Preparation of Coal-Based Graphene by Flash Joule Heating. ACS Omega.

[20] Li J., Luo L., Wang S., Song H., Jiang B. (2024). Recent advances in Joule-heating synthesis of functional nanomaterials for photo and electrocatalysis. PhotoMat.

[21] Wyss K.M., De Kleine R.D., Couvreur R.L., Kiziltas A., Mielewski D.F., Tour J.M. (2022). Upcycling end-of-life vehicle waste plastic into flash graphene. Commun. Eng..

[22] Wyss K.M., Beckham J.L., Chen W., Luong D.X., Hundi P., Raghuraman S., Shahsavari R., Tour J.M. (2021). Converting plastic waste pyrolysis ash into flash graphene. Carbon.

[23] Tour J. (2022). Conversion of Domestic US Coal into Exceedingly High-Quality Graphene.

[24] Jia C., Pang M., Lu Y., Liu Y., Zhuang M., Liu B., Lu J., Wei T., Wang L., Bian T. (2022). Graphene environmental footprint greatly reduced when derived from biomass waste via flash Joule heating. One Earth.

[25] Zhu X., Lin L., Pang M., Jia C., Xia L., Shi G., Zhang S., Lu Y., Sun L., Yu F. (2024). Continuous and low-carbon production of biomass flash graphene. Nat. Commun..

[26] Sattari K., Eddy L., Beckham J.L., Wyss K.M., Byfield R., Qian L., Tour J.M., Lin J. (2023). A scientific machine learning framework to understand flash graphene synthesis. Digit. Discov..

[27] Beckham J.L., Wyss K.M., Xie Y., McHugh E.A., Li J.T., Advincula P.A., Chen W., Lin J., Tour J.M. (2022). Machine Learning Guided Synthesis of Flash Graphene. Adv. Mater..

[28] Yap Y.W., Mahmed N., Norizan M.N., Rahim S.Z.A., Salimi M.N.A., Razak K.A., Mohamad I.S., Abdullah M.M.A.-B., Yunus M.Y.M. (2023). Recent Advances in Synthesis of Graphite from Agricultural Bio-Waste Material: A Review. Materials.

[29] Wyss K.M., Silva K.J., Bets K.V., Algozeeb W.A., Kittrell C., Teng C.H., Choi C.H., Chen W., Beckham J.L., Yakobson B.I. (2023). Synthesis of Clean Hydrogen Gas from Waste Plastic at Zero Net Cost. Adv. Mater..

[30] Sousa M.C., Buchanan J.W. (2000). Observational Models of Graphite Pencil Materials. Comput. Graph. Forum.

[31] McKendry P. (2002). Energy production from biomass (part 1): Overview of biomass. Bioresour. Technol..

[32] Vassilev S.V., Baxter D., Andersen L.K., Vassileva C.G. (2010). An overview of the chemical composition of biomass. Fuel.

[33] Kaza S., Yao L.C., Bhada-Tata P., Van Woerden F. (2018). What a Waste 2.0: A Global Snapshot of Solid Waste Management to 2050.

[34] Al-Salem S.M., Lettieri P., Baeyens J. (2009). Recycling and recovery routes of plastic solid waste (PSW): A review. Waste Manag..

[35] Anuar Sharuddin S.D., Abnisa F., Wan Daud W.M.A., Aroua M.K. (2016). A review on pyrolysis of plastic wastes. Energy Convers. Manag..

[36] Ververis C., Georghiou K., Christodoulakis N., Santas P., Santas R. (2004). Fiber dimensions, lignin and cellulose content of various plant materials and their suitability for paper production. Ind. Crop. Prod..

[37] Bajpai P. (2015). Generation of Waste in Pulp and Paper Mills. Management of Pulp and Paper Mill Waste.

[38] Paritosh K., Kushwaha S.K., Yadav M., Pareek N., Chawade A., Vivekanand V. (2017). Food Waste to Energy: An Overview of Sustainable Approaches for Food Waste Management and Nutrient Recycling. BioMed Res. Int..

[39] Xu F., Li Y., Ge X., Yang L., Li Y. (2018). Anaerobic digestion of food waste—Challenges and opportunities. Bioresour. Technol..

[40] Sjöström E. (1993). Wood Chemistry.

[41] Rowell R.M. (2012). Handbook of Wood Chemistry and Wood Composites.

[42] Wang Y. (2006). Recycling in Textiles.

[43] Pensupa N., Leu S.-Y., Hu Y., Du C., Liu H., Jing H., Wang H., Lin C.S.K. (2017). Recent Trends in Sustainable Textile Waste Recycling Methods: Current Situation and Future Prospects. Top. Curr. Chem..

[44] Tuinstra F., Koenig J.L. (1970). Raman Spectrum of Graphite. J. Chem. Phys..

[45] Ferrari A.C. (2007). Raman spectroscopy of graphene and graphite: Disorder, electron–phonon coupling, doping and nonadiabatic effects. Solid State Commun..

[46] Cançado L.G., Jorio A., Ferreira E.H.M., Stavale F., Achete C.A., Capaz R.B., Moutinho M.V.d.O., Lombardo A., Kulmala T.S., Ferrari A.C. (2011). Quantifying Defects in Graphene via Raman Spectroscopy at Different Excitation Energies. Nano Lett..

[47] Ferrari A.C., Basko D.M. (2013). Raman spectroscopy as a versatile tool for studying the properties of graphene. Nat. Nanotechnol..

[48] Childres I., Jauregui L.A., Park W., Cao H., Chen Y.P. (2013). Raman spectroscopy of graphene and related materials. New Dev. Photon Mater. Res..

[49] Ni Z.H., Wang H.M., Kasim J., Fan H.M., Yu T., Wu Y.H., Feng Y.P., Shen Z.X. (2007). Graphene Thickness Determination Using Reflection and Contrast Spectroscopy. Nano Lett..

[50] Beams R., Cançado L.G., Novotny L. (2015). Raman characterization of defects and dopants in graphene. J. Phys. Condens. Matter.

[51] Sheng S., Wu J.-B., Cong X., Li W., Gou J., Zhong Q., Cheng P., Tan P.-H., Chen L., Wu K. (2017). Vibrational Properties of a Monolayer Silicene Sheet Studied by Tip-Enhanced Raman Spectroscopy. Phys. Rev. Lett..

[52] Kalbac M., Reina-Cecco A., Farhat H., Kong J., Kavan L., Dresselhaus M.S. (2010). The Influence of Strong Electron and Hole Doping on the Raman Intensity of Chemical Vapor-Deposition Graphene. ACS Nano.

[53] Graf D., Molitor F., Ensslin K., Stampfer C., Jungen A., Hierold C., Wirtz L. (2007). Spatially Resolved Raman Spectroscopy of Single- and Few-Layer Graphene. Nano Lett..

[54] Kuryshchuk S., Kovalyuk T., Koziarskyi I., Solovan M. (2022). Structural, Electrical and Optical Properties of Graphite Films are Drawn with Pencils of Different Hardness. East Eur. J. Phys..

[55] Malard L.M., Pimenta M.A., Dresselhaus G., Dresselhaus M.S. (2009). Raman spectroscopy in graphene. Phys. Rep..

[56] Paton K.R., Varrla E., Backes C., Smith R.J., Khan U., O’Neill A., Boland C.S., Lotya M., Istrate O.M., King P. (2014). Scalable production of large quantities of defect-free few-layer graphene by shear exfoliation in liquids. Nat. Mater..

[57] Cançado L.G., Takai K., Enoki T., Endo M., Kim Y.A., Mizusaki H., Jorio A., Coelho L.N., Magalhães-Paniago R., Pimenta M.A. (2006). General equation for the determination of the crystallite size La of nanographite by Raman spectroscopy. Appl. Phys. Lett..

[58] Lee D.S., Riedl C., Krauss B., von Klitzing K., Starke U., Smet J.H. (2008). Raman Spectra of Epitaxial Graphene on SiC and of Epitaxial Graphene Transferred to SiO_2_. Nano Lett..

[59] Ramamoorthy H., Buapan K., Chiawchan T., Thamkrongart K., Somphonsane R. (2021). Exploration of the temperature-dependent correlations present in the structural, morphological and electrical properties of thermally reduced free-standing graphene oxide papers. J. Mater. Sci..

[60] Das A., Pisana S., Chakraborty B., Piscanec S., Saha S.K., Waghmare U.V., Novoselov K.S., Krishnamurthy H.R., Geim A.K., Ferrari A.C. (2008). Monitoring dopants by Raman scattering in an electrochemically top-gated graphene transistor. Nat. Nanotechnol..

